# A Comparative Study of Cell Specific Effects of Systemic and Volatile Anesthetics on Identified Motor Neurons and Interneurons of *Lymnaea stagnalis* (L.), Both in the Isolated Brain and in Single Cell Culture

**DOI:** 10.3389/fphys.2019.00583

**Published:** 2019-05-31

**Authors:** Hadi Fathi Moghadam, Talay Yar, Munir M. Qazzaz, Ibrahim Abdelrazig Ahmed, William Winlow

**Affiliations:** ^1^Department of Physiology, Physiology Research Centre, Faculty of Medicine, Ahvaz Jundishapur University of Medical Sciences, Ahvaz, Iran; ^2^Department of Physiology, College of Medicine, Imam Abdulrahman Bin Faisal University, Dammam, Saudi Arabia; ^3^Faculty of Pharmacy, Nursing, and Health Professions, University of Birzeit, Birzeit, Palestine; ^4^Faculty of Medicine, University of Garden City, Khartoum, Sudan; ^5^Department of Biology, University of Naples Federico II, Naples, Italy; ^6^Institute of Ageing and Chronic Diseases, University of Liverpool, Liverpool, United Kingdom; ^7^NPC Newton, Preston, United Kingdom

**Keywords:** systemic anesthetics, volatile anesthetics, *Lymnaea stagnalis*, identified motor neurons and interneurons, paroxysmal depolarization shifts, action potentials

## Abstract

1. A comparative descriptive analysis of systemic (sodium pentobarbital, sodium thiopentone, ketamine) and volatile (halothane, isoflurane, enflurane) general anesthetics revealed important differences in the neuronal responses of identified motor neurons and interneurons in the isolated central nervous system (CNS) and cultured identified neurons in single cell culture of Lymnaea stagnalis (L.).

2. At high enough concentrations all anesthetics eventually caused cessation of spontaneous or evoked action potentials, but volatile anesthetics were much faster acting. Halothane at low concentrations caused excitation, thought to be equivalent to the early excitatory phase of anesthesia. Strong synaptic inputs were not always abolished by pentobarbital.

3. There were cell specific concentration-dependent responses to halothane and pentobarbital in terms of membrane potential, action potential characteristics, the after hyperpolarization and patterned activity. Individual neurons generated specific responses to the applied anesthetics.

4. The inhalation anesthetics, enflurane, and isoflurane, showed little concentration dependence of effect, in contrast to results obtained with halothane. Enflurane was faster acting than halothane and isoflurane was particularly different, producing quiescence in all cells types studied at all concentrations studied.

5. Halothane, enflurane, the barbiturate general anesthetics, pentobarbital, and sodium thiopentone and the dissociative anesthetic ketamine, produced two distinctly different effects which could be correlated with cell type and their location in the isolated brain: either a decline in spontaneous and evoked activity prior to quiescence in interneurons or paroxysmal depolarizing shifts (PDS) in motor neurons, again prior to quiescence, which were reversed when the anesthetic was eliminated from the bath. In the strongly electrically coupled motor neurons, VD1 and RPD2, both types of response were observed, depending on the anesthetic used. Thus, with the exception isoflurane, all the motor neurons subjected to the anesthetic agents studied here were capable of generating PDS *in situ*, but the interneurons did not do so.

6. The effects of halothane on isolated cultured neurons indicates that PDS can be generated by single identified neurons in the absence of synaptic inputs. Further, many instances of PDS in neurons that do not generate it *in situ* have been found in cultured neurons. The nature of PDS is discussed.

## Introduction

In a recent review (Winlow et al., 2018) we suggested that clues to the likely effects of anesthetics on cephalopods such as *Octopus vulgaris* might be gained from related studies on gastropod molluscs such as *Lymnaea stagnalis* which has proved to be an excellent model system for studies of the cellular actions of general anesthetics on individual identified neurons (e.g., McCrohan et al., 1987; Winlow et al., 1987, 1995; Franks and Lieb, 1988; McKenzie et al., 1995; Spencer et al., 1995, 1996) many of which have known functions. The utility of such a preparation is that it allows us to study of the modes of action of general anesthetics, since behavioral, cell physiological, and biophysical experiments can all be performed on this preparation (e.g., McCrohan et al., 1987; Girdlestone et al., 1989a,b; Winlow et al., 2018) at clinical concentrations (Girdlestone et al., 1989c; Yar and Winlow, 2016b). Such studies have provided good basic information on the modes of action of general anesthetics and are also being used to underpin related studies on the actions of volatile anesthetics on cephalopod molluscs such as *Octopus vulgaris* (Polese et al., 2014; Winlow et al., 2018). Whole cell patch clamp studies on the effects of halothane on calcium currents have been reported elsewhere (Yar and Winlow, 2016a,b) as have studies on passive properties of the electrically coupled giant neurons VD1 and RPD2 (Qazzaz and Winlow, 2015, 2017) in response to halothane, isoflurane, and pentobarbitone.

General anesthetics vary widely in structure and physico-chemical properties, but in spite of this diversity they all have the same eventual physiological effect, depression of neuronal activities in the central nervous system culminating in loss of perception and consciousness, due to either axonal conduction block, or diminution of both excitatory (Richards, 1983), and inhibitory (Spencer et al., 1996) synaptic transmission. In addition, neurons have very diverse morphologies, physiologies, biochemical, and pharmacological properties so they might be expected to have differing responses to applied agents and this is the case in relation to anesthetic substances applied to identified neurons of the pond snail *Lymnaea stagnalis* (L.) (Winlow et al., 1991, 1992, 1995, 2018; Qazzaz and Winlow, 2015, 2017).

Previous experiments on *Lymnaea* with menthol demonstrated that some neurons became quiescent and that others generated paroxysmal depolarizing shifts (PDS) in its presence (Haydon et al., 1982) and comparable actions have previously been described in preliminary reports on isolated identified neurons in culture (Winlow et al., 1992; Yar, 1992). Some neurons became quiescent immediately, whilst others only do so after exhibiting PDS as demonstrated for halothane (Girdlestone, 1986; Winlow et al., 1992). PDS is generated by a large depolarizing wave (Jefferys, 2010) which may be endogenous or synaptically driven, and which may trigger a series of damped action potentials. The original experiments on *Lymnaea* anesthesia were carried out using menthol (Haydon et al.) which is slow acting and causes distress to the animal. Since 1982 further studies have been carried out on clinical analgesics and anesthetics on various molluscan preparations (see Winlow et al., 2018 for review). The neurons studied here may be divided into motor neurons and interneurons (see Discussion) and the actions of the anesthetics have rather different effects on these neuron types. Their functions are reviewed in detail elsewhere (Winlow and Polese, 2014).

This is the first report to collate, compare, and contrast all our previously unpublished data on the electrophysiological actions of both systemic and volatile inhalational anesthetic agents on the discharges and patterning of identified *Lymnaea* motor neurons and interneurons, both *in situ* in the intact isolated brain and in single cell culture. Here, we present a descriptive comparative analysis of the actions of the anesthetics on neural discharges. We also present statistical comparison of the modifications of spike width and amplitude of neurons grown in culture as compared with those *in situ* and demonstrate their responses to applied anesthetics.

## Materials and Methods

Experiments were carried out on *Lymnaea stagnalis* obtained from suppliers (Blades Biological, Kent), maintained in circulating aerated tap water at room temperature and fed on lettuce supplemented with fish food. Snails of 1–4 g in weight were chosen for dissection and were transferred to a dissecting dish coated with Sylgard resin (Dow Corning, GmbH, USA). Dissection was carried out at 20°C in HEPES buffered saline (HBS) buffered to pH 7.8 with NaOH as previously described (Benjamin and Winlow, 1981).

### Experiments on Neurons in the Intact Brain

**Figure 1:**
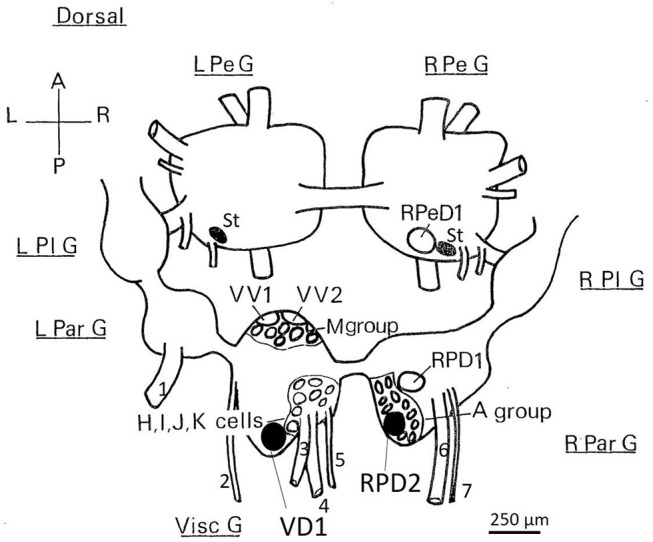
Dorsal view of the location of the somata of *Lymnaea* neurons utilized in this study. RPeD1, RPD1 are interneurons and VV1/VV2 is a putative interneuron, while VD1/RPD2, A group, M group, and the J cells are motor neurons—neither soma size nor color can be used to discriminate these neuronal types. Shaded cell bodies are white, unshaded cell bodies are orange. The diagram is of the dorsal surface of the left parietal ganglion (L Par G, from which the left parietal nerve−1, emerges), the median visceral ganglion (Visc G from which the cutaneous pallial nerve−2, the intestinal nerve−3, the anal nerve−4, and the genital nerve−5 emerge), the right parietal ganglion (R Par G from which the right internal parietal nerve−6 and the right external parietal nerve−7 emerge), the pleural ganglia (L Pl G and R Pl G) and the pedal ganglia (L Pe G and R Pe G). For further details see Slade et al., 1981. L, left; R, right; A, anterior; P, posterior, St: statocysts.

The recording chamber was regularly flushed with fresh aerated HBS, barbiturate saline or saline equilibrated with volatile anesthetics during the course of the experiment. The neurons were identified under binocular magnification according to their color, locations and electrical activity (Winlow and Benjamin, 1976; Benjamin and Winlow, 1981; Winlow et al., 1981). Intracellular recordings were made from individual motor neurons and interneurons (see Figure 1) using filamented glass borosilicate glass microelectrodes (Clark ElectroMedical Instruments), filled with saturated K_2_SO_4_, with a resistance of 10–30 MΩ. Results were obtained using standard electrophysiological techniques, including Neurolog NL 101 bridge-balanced amplifiers and appropriate stimulators. Signals were monitored on a Tektronix oscilloscope and via a CED interface connected to an EPSON PCV computer for subsequent analysis. The CED SPIKE2 programme was used to capture and process the data.

### Data Analysis

Data analysis of the discharges of individual cultured neurons were performed according to Yar et al. (1993). Statistical analysis of significance using Student's *t*-tests were performed as appropriate using the “Oxstat” program (Oxford Logic from Wallingford Computer Services, Oxfordshire, OX10 9BJ). Where possible, data were expressed as mean ± standard deviation (SD) and values were considered statistically significant at a confidence level of 95%. This was not appropriate with the patterned activity presented in most of the Figures, where typical traces, based on long-term recordings are presented. Subgroups of the total number of neurons investigated were studied to reveal changes in their discharge patterns in the presence of applied anesthetics. Temporal changes in discharge patterns associated with anesthetics were clear and the total number of observations of this type of activity are indicated in both the text and figure legends.

### Systemic Anesthetics

The standard protocol used for standard electrophysiology with pentobarbital (a relatively slow onset and relatively long duration barbiturate−28) was to:

1. record the normal activity of the cell in normal HEPES saline;
2. perfuse the preparation with the low concentration of pentobarbital (1 mM) for at least 30 min (maximum 1 h), and make recordings at 6 min intervals;
3. perfuse the preparation with the high concentration of pentobarbital (2 mM) for another 30 min (maximum 1 h), and make recordings every 6 min;
4. wash the preparation continuously with saline for 1 h and make recordings at 10 min intervals.

Variations on this protocol were used as required, usually in the form of square depolarizing current pulses in order to stimulate otherwise quiescent cells. The effects of Thiopentone sodium 0.5 mM (May and Baker), a rapid onset, brief duration barbiturate (Bowman and Rand, 1980), and Ketamine hydrochloride (Parke-Davis), 0.2 mM were also studied on small numbers of individual neurons using similar methods.

### Volatile Anesthetics

The volatile anesthetics were delivered to the isolated CNS preparation through a specially designed and carefully controlled anesthetic delivery system as previously described for ED_50_ determinations (Girdlestone et al., 1989c) or using standard anesthetic vaporizers (Ohmeda). Concentrations of vaporized halothane, enflurane, and isoflurane, between 0.25 and 4.00% v.v., were monitored using a commercial anesthetic monitor (Datex Normac AA-102) and equilibrated with HEPES buffered saline (HBS) which was then gravity fed to superfuse the preparation at a flow rate of 60 ml.min^−1^.

### Experiments on Identified, Isolated Neurons in Culture

Preparation and incubation of neurons as rounded cells without neuritic extensions in short term culture, for 1 to 2 days, was carried out according to the methods of Yar (Yar, 1992), Walcourt-Ambakederemo and Winlow (Walcourt-Ambakederemo and Winlow, 1994), Ahmed et al. (Ahmed et al., 1997), and Yar and Winlow (Yar and Winlow, 2016a) and anesthetics were applied as above.

## Results

All the neurons tested with the various anesthetics used in this study eventually became quiescent if an adequate concentration of anesthetic was applied to the brain or to isolated, cultured, identified neurons for an appropriate length time, which varies from one cell type to another. The pathways to silence differ in that some neurons gradually become quiescent, while others exhibit a series of paroxysmal shifts (PDS) prior to quiescence and yet others exhibit both types of response depending on the anesthetic used.

### Experiments on Neurons in the Intact, Isolated Brain

#### Cell Specific Effects of Systemic Anesthetics

**Figure 2:**
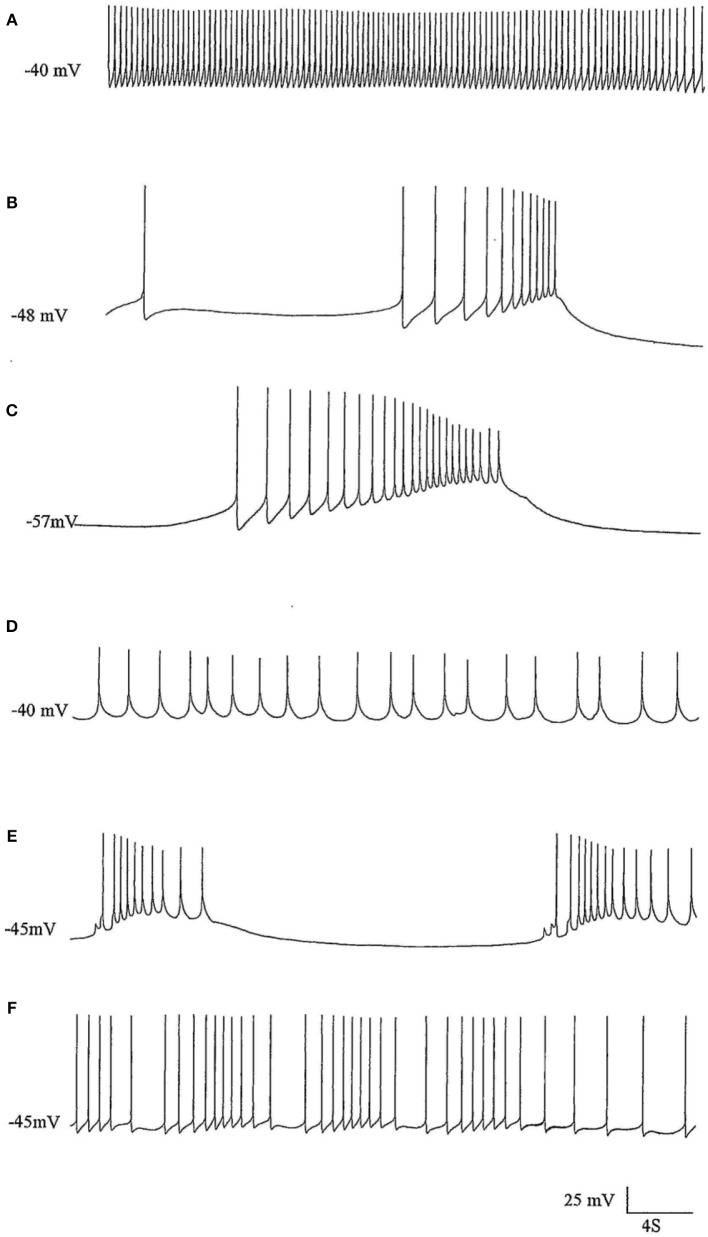
The effects of pentobarbital on the spike pattern of a J cell motor neuron (*n* = 4). **(A)** Control; **(B)** 30 min after application of 1 mM pentobarbital; **(C)** 2 mM pentobabbital after 6 min; **(D)** 2 mM pentobarbital after 18 min; **(E)** 2 mM pentobarbital after 30 min; **(F)** 20 min after continuous washout with fresh HBS. Note that pentobarbital induces hyperpolarisation which is reversible with continuous washing and the large depolarisations induce damped action potentials associated with 1 PDS in B,C, and E and that the cell is depolarised back to control levels in D, but with reduced spike amplitude and ferquency and loss of AHP.

**Figure 3:**
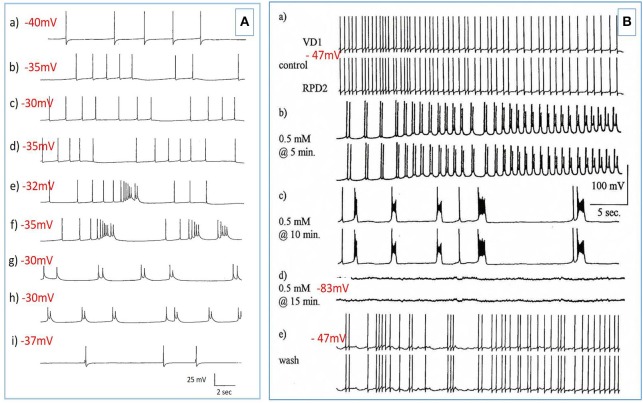
PDS induced by superfusion of the brain with Pentobarbital. **(A)** The effect of Pentobarbital on the pattern and frequency of an M group motor neuron (*n* = 4). **(a)** Control; **(b)** after 6 min in 1 mM Pentobarbital; **(c)** after 12 min; **(d)** after 18 min; **(e)** after 24 min; **(f)** after 30 min; 2 mM pentobarbital **(g)** after 6 min; **(h)** after 30 min; **(i)** 40 min continuous wash in normal saline. Membrane potential decreased with application of both concentrations of pentobarbital and was partially reversible with continuous wash after 40 min. **(B)** Effect of 0.5 mM pentobarbital on the spontaneous discharge of the strongly electrically coupled motor neurons VD1 and RPD2 (*n* = 8). **(a)** Normal electrical activity in VD1 and RPD2 at a resting potential of −47 mV. **(b)** 5 min after perfusing the brain with 0.5 mM pentobarbital in HBS the neurons started to fire in doublets and then **(c)** gradually hyperpolarized and developed PDS, after which **(d)** they became quiescent at a membrane potential of −83 mV. **(e)** The effect of pentobarbital was completely reversed after washing in clean HBS and the cells returned to their normal discharge.

**Figure 4:**
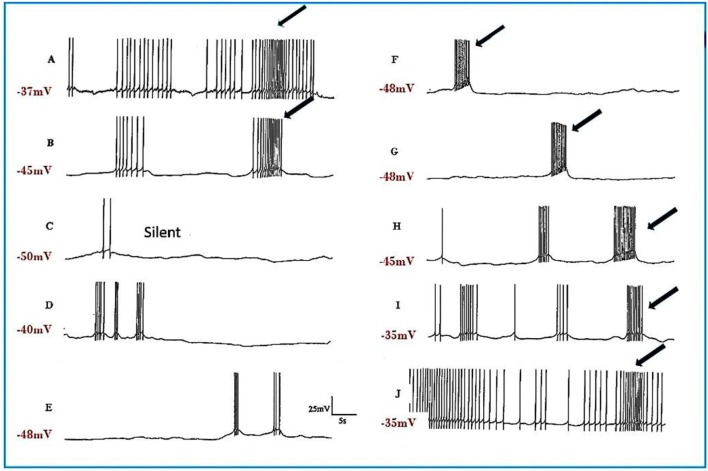
Pentobarbital suppresses the spontaneous patterned discharge of interneuron RPeD1 (*n* = 6), but does not suppress the powerful spontaneous synaptic input from the input 3 interneuron (arrows), which is also part of the respiratory central pattern generator and is located in the right parietal ganglion (Syed et al., 1990; Winlow and Polese, 2014). **(A)** Normal; **(B)** 1 mM pentobarbital after 6 min; **(C)** after 12 min which was silent until **(D)** after 30 min; **(E)** 2 mM pentobarbital after 18 min; **(F)** after 24 min; **(G)** after 30 min; **(H)** after 10 min washout; **(I)** after 20 min; **(J)** after 30 min; (M) after 40 min. Application of pentobarbital hyperpolarized RPeD1. Continuous wash out of pentobarbital decreased membrane potential of RPeD1 to normal with a resumption of normal patterned activity.

**a) Pentobarbital** induces PDS in the motor neurons studied here (*n* = 109), but not in interneurons (*n* = 41). Every cell tested had remained stable for at least 10 min following penetration. Pentobarbital, at both the concentrations used, caused the membrane to either hyperpolarize or depolarize and either reduced or abolished the after hyperpolarization (AHP) (Table 1) when first added to the bath and then usually caused PDS (Figures 2, 3), quiescence or significant suppression of normal patterned activity (Figure 4A), depending on the cell type (as summarized in Table 2.

**Table 1:** Characterization of effects of 1–2 mM pentobarbital Increases due to fall in conductance and 1–2% halothane on spontaneous activity of identified *Lymnaea* neurons (*n* = 110).

	**Dose Dep**.	**Motor neurones exhibiting PDS before quiescence**			**Interneurons with dual responses**		**Interneurons usually tending to quiescence**		
**Cell type**		**J cells**	**M group**	**A group**	**VD1**	**RPD2**	**VV1/2**	**RPeD1**	**RPD1**
A P type		1	2	2	2	2	2	Usually 2	2
E_m_-Pentobarbital	X	Hyp	Dep	Hyp	Substantial Hyp ca. 30–35 mV after PDS		Hyp	Hyp, ca 10–12 mV	Hyp
E_m_-Halothane	X	Minimal Hyp (a few mV)			Substantial Hyp ca. 30–35 mV (see Qazzaz and Winlow, 2015)		Minimal variable effects (a few mV)–Hyp, Dep or no change		
APA-Pentobarbital	√	Declining amplitude during PDS bursts, then quiescence			Normal amplitude gradually declining with onset of PDS		Gradual decline	No change	Declines, slow recovery
APA-Halothane	√	APA largely maintained prior to PDS			Normal APA, then sudden quiescence		Reduced APA		
AP Duration-Pentobarbital and Halothane	√	Decreases	Generally decreased, mainly due to diminution of the calcium dependent pseudoplateau in neurons with type 2 action potentials						
AP Frequency-Pentobarbital	√	Cells become quiescent and then PDS is induced	AP frequency initially increases and is eventually replaced by PDS, which reduces to doublets and triplets at higher doses	Frequency declines and APs replaced by irregular dose-dependent PDS. Long recovery time	Gradual decline and then onset of doublet firing prior to PDS		Gradual decline	Gradual decline, but strong input 3 often apparent	Rapid, long-lasting quiescence. Long recovery time
AP Frequency-Halothane	√	Gradual decline of spontaneous activity into quiescence			Immediate quiescence accompanied by strong epsps. Recovers quickly		Gradual reduction in discharge frequency	Prolonged inhibition of APs but with attendant strong epsp inputs	Strong and immediate early excitation and then quiescence
		Early excitatory response of variable duration depending on neuron type, then frequency declines							
AHP-Pentobarbital	√	Abolished			Markedly reduced		Reduced	Minimal reduction	Reduced
AHP-Halothane	√	Reduced			Minimal change		Markedly reduced		

R_m_-Pentobarbital	X	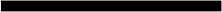			Increases due to fall in dose -independent **g**_**m**_ (see Qazzaz and Winlow, 2015)		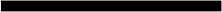		
R_m_-Halothane	√	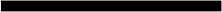			Declines due to rise in dose –dependent g_m_ (and see Qazzaz and Winlow, 2015, re VD1/RPD2)			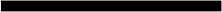	

*E_m_, membrane potential; AHP, afterhyperpolarization; AP, action potential; APA, action potential amplitude; Hyp, hyperpolarization; Dep, depolarization; Dose dep, dose dependency, X = no dose-dependency. Data on input resistance (R_m_) of neurons is incomplete, but relevant to the present study; g_m_ , membrane conductance*.

**Table 2:** Differential actions of volatile and systemic anesthetics on specific cells and cell groups (see Figure 1) in the isolated brain of *Lymnaea*.

	**Motor neurons usually exhibiting PDS**					**Motor and interneurons usually tending to Quiescence**					
**Cell type**	**A gp**	**J cells**	**M gp**	**RPD2**	**VD1**	**VD1**	**RPD2**	**VV1/2**	**RPeD1**	**RPD1**	**Total *n* by agent**
AP type (1 or 2)	2	1	2	2	2	2	2	2	Mostly 2	2	
Halothane	8	6	4			22	20	6	11	7(1)	85
Enflurane	1	1	1					2(1)	2	1	9
											
Isoflurane	(2)	(2)	(1)			22	25	1	2	2	57
Pentobarbital	14	13	18	32	32			10	16	15	150
Thiopentone	2		2					(1)		1	6
Ketamine	2	(1)	1							2	6
Total n by cell type	27(2)	20 (3)	26(1)	32	32	44	45	19 (2)	31	28 (1)	**313**
% cells with PDS or quiescence	**93.1**	**87.0**	**96.3**	**100**	**100**	**100**	**100**	**90.5**	**100**	**96.6**	

*n = number of cells studied in each case. The green box at bottom right shows the total number of individual neurons studied. In isoflurane (orange row) all the cells tested (n = 57) became quiescent. Numbers in brackets indicate that some cells exhibited either PDS or quiescence, unlike the majority of that cell type. Unlike other cells the motor neurons VD1 and RPD2 (blue columns) always exhibited PDS in Na pentobarbital, but did not do so in halothane*.

#### Motor Neurons Usually Exhibiting PDS

**Figure 5:**
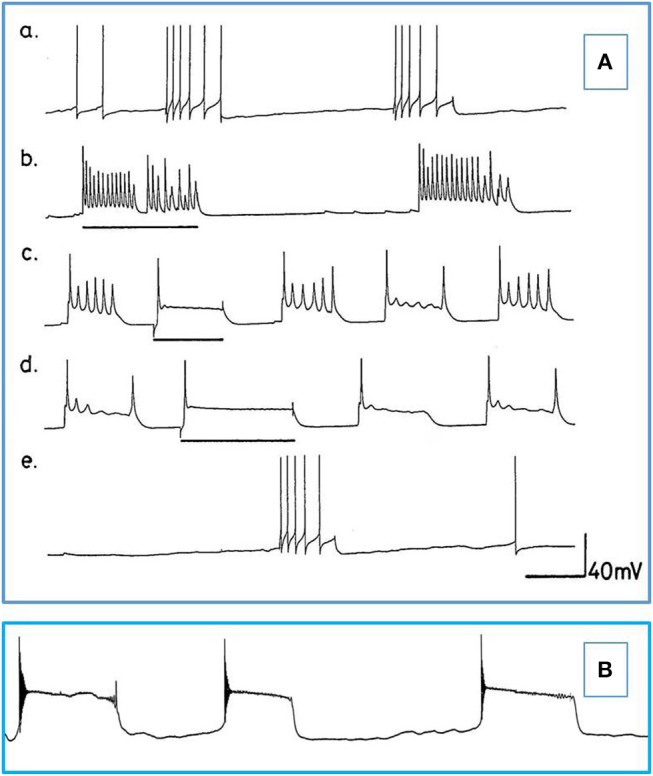
Actions of barbiturates on motor neurons **(A)** Effects of thiopentone on spontaneous and evoked activity of A group motor neurons (*n* = 2 and 2 M group cells exhibited similar activity). Depolarizing current pulses are indicated by horizontal bars. **(a)** Normal activity. **(b–d)** Evoked and spontaneous PDS at 1, 4, and 7 min, respectively after addition of thiopentone. **(e)** Recovery after 15 min in fresh HBS. Time calibrations: a, b, c and e 5 s; d 10 s. **(B)** An example of spontaneous, repetitive PDS in a J cell motor neuron in the presence of 2 mM pentobarbital. The cell had not been artificially hyperpolarized. Time calibration 5 s.

The J cells (*n* = 13) of the right parietal ganglion always exhibited PDS in the presence of pentobarbital, as do the neurons of A group (*n* = 13) (Figure 2) and M group (*n* = 18) of the visceral ganglion (Figure 3A). The underlying depolarizing wave associated with PDS is particularly clear in Figure 2C and occurred spontaneously for several minutes in J cells before quiescence in the presence of 2 mM pentobarbital (Figure 5B). In addition the strongly electrically coupled motor neurons VD1 and RPD2 also exhibited PDS in the presence of pentobarbital (Figure 3B) (*n* = 8) and were different from other neurons reported here in that they became quiescent in halothane as well as in isoflurane (14 and see below). VD1 and RPD2 eventually become quiescent in 0.5 mM pentobarbital and are strongly hyperpolarized under these circumstances. Such major shifts in membrane potential (E_m_) have not been observed in the other identified neurons studied here.

#### Interneurons Usually Exhibiting a Tendency to Quiescence

**RPeD1** (*n* = 7): Application of pentobarbital (1 mM) to RPeD1 causes a gradual reduction in spontaneous firing (Figure 4) as the cell hyperpolarizes. The data showed RPeD1 to fire spontaneously and irregularly under normal circumstances; because it receives powerful synaptic input from the input 3 interneuron (see Figure 4). Continuous application of 1 mM pentobarbital always induced hyperpolarization and suppressed the spontaneous pattern of firing but could not block EPSPs in this cell. The data showed that in 2 mM pentobarbital, the membrane potential increased, and excitability decreased (Figure 4F). Continuous wash out of pentobarbital increased the frequency of firing and the pattern of neuronal activation started to return to normal (Figures 4A,I). After 40–60 min continuous washing in normal saline, the spontaneous pattern of firing always returned to normal (Figure 4J).
**VV1/VV2** (*n* = 6) showed a gradual decline to quiescence in pentobarbital, which slowly returned to normal after washout.
**RPD1** (*n* = 5) is much more sensitive to pentobarbital as compared with the other cells studied here. Application of 1 mM pentobarbital induced immediate and long- lasting quiescence (over 30 min) with a clear hyperpolarization and a long recovery period of over 1 h following washout.
**b) Thiopentone** Only 6 experiments were carried out with 0.5 mM thiopentone, but the cells studied responded in a similar way to those in pentobarbital (Figure 5A and Table 2), except that VV1/2 exhibited PDS. Recovery was much more rapid than in pentobarbital as would be expected from this rapid onset brief duration anesthetic (Rang et al., 2007).
**c) Ketamine** A further 6 cells were observed (2 × A group, 2 × RPD1, an M group cell and a J cell) after addition of ketamine, but again cell specific effects were observed as summarized in Table 2. Unusually the J cell did not exhibit PDS. Again recovery was more rapid than in thiopentone.

#### Cell Specific Effects of Volatile Anesthetics

The effects of halothane, isoflurane, and enflurane on identified neurons in the isolated CNS of *Lymnaea* (*n* = 151) were characterized at concentrations between 0.5 and 4.0% v/v.

#### Actions of Halothane on Interneurons and the Giant Motor Neurons VD1 and RPD2

**Figure 6:**
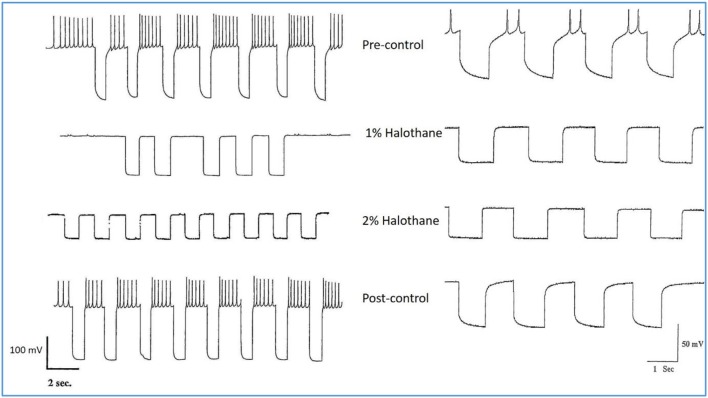
Action of halothane on the input resistance on two examples of motor neuron VD1. 0.1 nA pulses of 1.0 S duration were injected into the cells via the bridge-balanced recording electrode. In both cases the pulse was seen to diminish in a dose dependent manner in halothane, indicating a fall in membrane input resistance (R_m_) of the cells and hence an increase in conductance as halothane concentration increased. The left hand cell was maintained at normal resting potential, while the right hand cell had been hyperpolarized by a few millivolts, very close to threshold. Similar data were obtained from RPD1 and VV1/VV2. N.B. A detailed statistical analysis of the effects of anesthetics on the passive membrane properties of VD1/RPD2 appears elsewhere (Qazzaz and Winlow, 2017).

Experiments on input resistance were carried out on the putative interneurons VV1/VV2 (*n* = 6) and on the strongly electrically coupled motor neurons VD1 and RPD2 (*n* = 18) to determine the effects of halothane on membrane conductance. There was a clear concentration-dependent increase in conductance due to a decline in input resistance both in halothane (Figure 6) and isoflurane.

**Figure 7:**
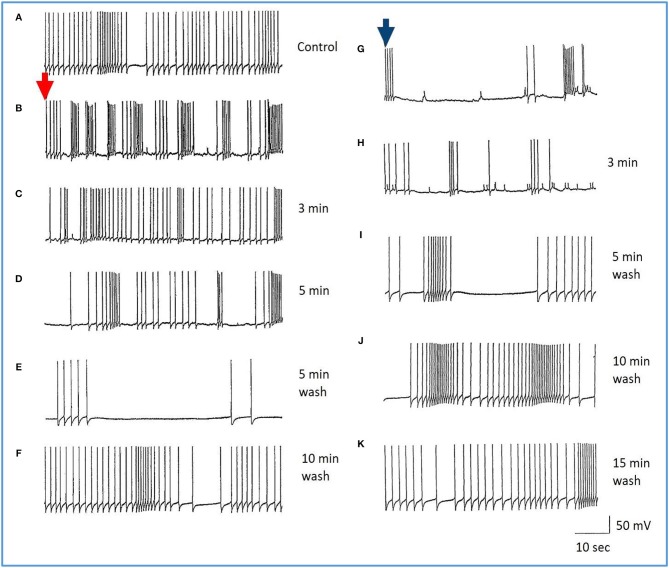
Common responses and concentration dependence of neurons at low and elevated halothane concentrations demonstrated in motor neuron RPD2 (*n* = 5). **(A)** Irregular spontaneous discharge; **(B)** Addition of 0.5% halothane (red arrow) generated enhanced patterning of the discharge and then a gradual decline of the discharge rate with time **(C,D)** after which the preparation was washed in clean saline **(E,F)** and 1% halothane was added at **(G)** (blue arrow). The neuron eventually became quiescent at the end of trace **(H)** and was then washed **(I–K)** returning to a normal discharge pattern after 15 min in HBS **(K)**. The spike amplitude and AHP were diminished in both 0.5% **(C,D)** and 1% halothane **(G,H)**.

Different concentrations of halothane, produced dramatic, concentration-dependent effects which can be generalized for the following cell types (*n* = 85). At low concentrations (0.25–1%) the interneurons RPeD1 (*n* = 11), VV1/2 (*n* = 6), RPD1 (*n* = 20) and the giant motor neurons VD1 (*n* = 22) and RPD2 (*n* = 20) share certain common responses, in spite of variability between neuron types. Sub populations of these neurons were therefore studied in detail to reveal the changes in their discharge patterns to applied anesthetics. Here the responses of RPD2 are used as the exemplar of the common responses to 0.5 and 1.0% halothane (Figure 7) which are characterized by an initial increase in the patterned discharge activity, perhaps the cellular equivalent of the early excitatory phase of anesthesia observed by Guedel (1937). This is followed by a period of diminished spontaneous action potential frequency and/or their complete cessation. Also, there was a marked reduction in the action potential amplitude and the amplitude of the AHP (Figures 7B–D,G,H). Higher concentrations of halothane (0.75-2.00%), i.e., those causing loss of the withdrawal response in 68-100% of animals) (Girdlestone et al., 1989c), caused a general depressant effect on the spontaneous activity of all the neurons examined.

**Figure 8:**
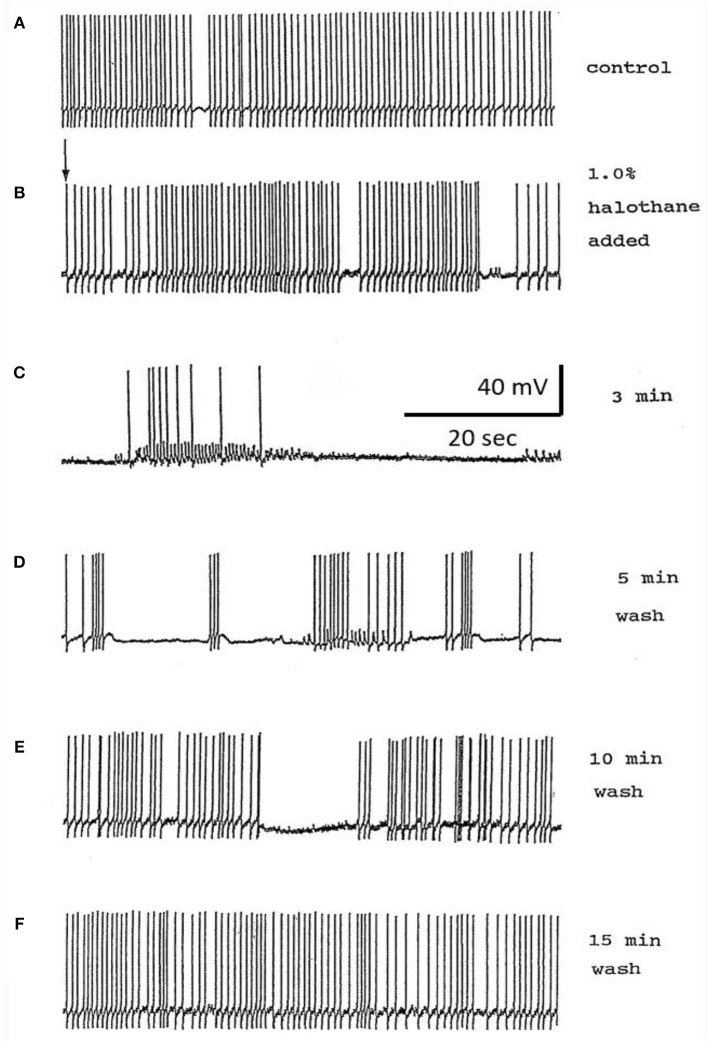
Effects of 1.0% halothane on the interneuron RPeD1 (*n* = 7). **(A)** Control; **(B)** 1.0% halothane was added at arrow. Generally there was a slight reduction in the discharge frequency as well as in the depth of AHP upon perfusion with halothane **(C)** shows the response of the cell after 3 min of application of halothane. The cell was largely inhibited and became quiescent as the cell hyperpolarized after the last spike shown here, but with a large number of e.p.s.ps., due to many synaptic inputs which failed to develop into full action potentials. **(D–F)** show the effects of rinsing the preparation continuously with normal saline for 5, 10, and 15 min, respectively. The cell partially recovered to control levels, in terms of the spontaneous discharge rate and the amplitudes of the AP and AHP.

In the VV1/2 (*n* = 6) and RPD1 (*n* = 5) neurons, the excitatory period was found to persist as long as the preparation was continuously perfused with low (0.5–0.75%) concentrations of halothane until the beginning of washing, whereas, in RPeD1(*n* = 7) and RPD2(*n* = 5) neurons, the excitatory period tended to be much briefer, lasting only a few minutes before quiescence. For example, in 1.0% halothane RPeD1 neurons became quiescent in just over 3 min (Figure 8C) as did RPD1. All the studied neurons showed a substantial reduction in the action potential amplitude and the amplitude of the AHP after perfusion with 0.5% halothane, but some neurons seem to be more affected than others. For instance, the AHP of RPD1 neurons (*n* = 5) was completely abolished in 1% halothane, whereas, RPeD1 neurons (Figure 8) were the least affected in this respect, in common with the effects of pentobarbital upon them(Figure 4). The effects of 0.5–1.0% halothane on the different neurons were found to be reversible following a 10–15 min wash in the majority of cases, except that all examples of RPD1, took over an hour to recover. The resting membrane potential (E_m_) of these neurons was in the range of −40 to −50 mV. Application of halothane was found to produce a variable effect on the E_m_ in the studied neurons (*n* = 23), depending on their initial E_m_. Sometimes, halothane depolarized the neuron (*n* = 8), or hyperpolarized the neuron (*n* = 11) by a few mV and sometimes there was no marked effect on the E_m_ (*n* = 4).

#### Actions of Halothane on the Smaller Motor Neurons

**Figure 9:**
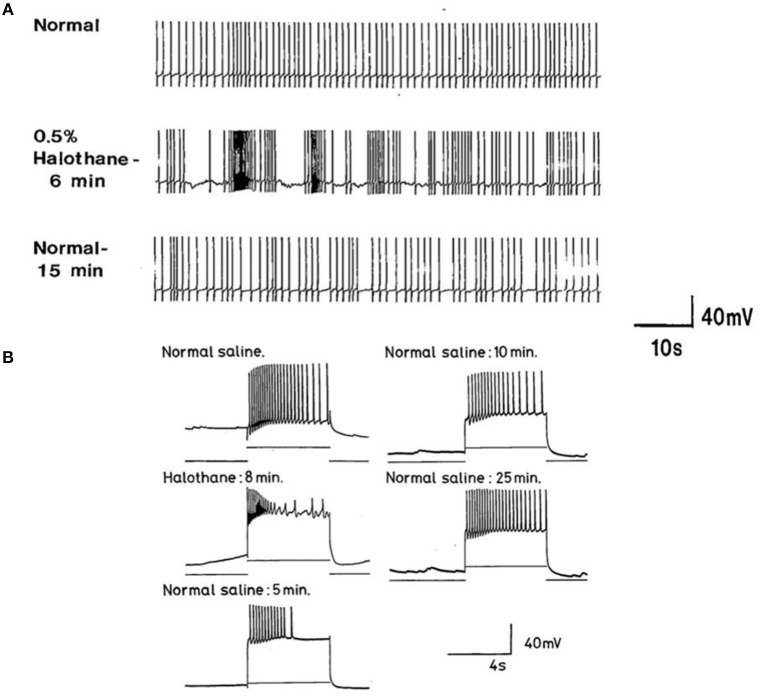
Concentration dependence of the effects of halothane on A group motor neurons (*n* = 8). **(A)** a low dose of halothane (0.5 %) caused patterning of an A cell spontaneous discharge, demonstrated by the occurrence of PSPs and high frequency bursting activity. **(B)** In 2% halothane neuronal activity of another A cell was completely suppressed, but PDS could still be elicited by depolarizing stimuli (1–2 nA for 5 S) into the neuronal soma. In both cases there was complete recovery on rinsing in HBS.

The actions of halothane on the smaller motor neurons (A group, *n* = 8, M group, *n* = 4, and J cells, *n* = 6) are exemplified in Figure 9 where the patterned activity of an a group neuron is enhanced in 0.5% halothane (Figure 9A) and remained so until washout after which normal activity in all these cells resumed within 15 min. In 2% halothane, all cells initially generated PDS, but eventually all became silent, although PDS could be evoked by depolarizing current pulses (Figure 9B).

#### Concentration-Dependent Effects of Halothane on Evoked Responses

**Figure 10:**
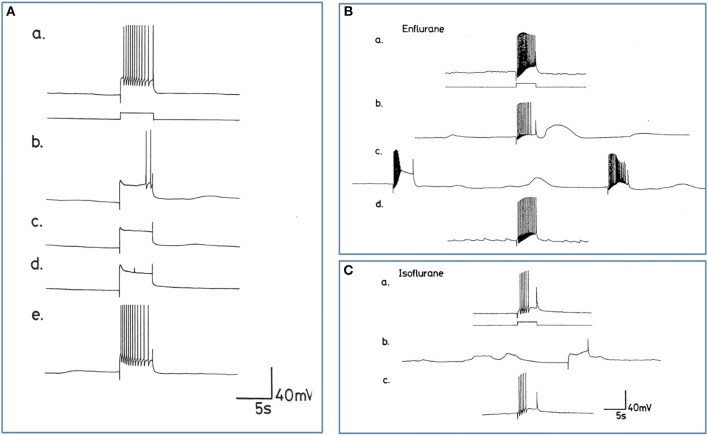
Differential actions of enflurane and isoflurane on individual identified neurons. **(A)** Effect of 1% enflurane on evoked activity in interneuron RPD1. **(a)** Depolarizing current pulses of 1 nA and 5 s duration evoked a train of spikes in normal saline. **(b)** After 30 s in enflurane, the frequency of evoked action potentials decreased, until **(e)** the normal evoked activity was restored 10 min after rinsing. **(B,C)** Large amplitude depolarization's were seen in the presence of enflurane and isoflurane when action potentials were evoked as in box 1. In **(Ba)** a train of action potentials was evoked in an M group motor neuron in HBS. **(b)** After 3 min in 2.0% enflurane, action potentials could still be evoked and a large amplitude, subthreshold depolarization occurred spontaneously. **(c)** After 4 min of superfusion the large amplitude depolarization was still present, but each of the depolarizing current pulses now triggered PDS, rather than a normal spike train. **(d)** There was full recovery of the evoked response after rinsing in HBS. In **(C)**, another example of the RPD1 interneuron is shown, in which both spontaneous and evoked effects are seen in the presence of isoflurane **(b)**, again accompanied by large depolarizing potentials, but no action potentials. **(a,c)** are pre and post c-controls.

Since all types of neurons eventually become silent at concentrations of volatile anesthetics >1.0%, an investigation of the comparative responses of interneurons and motor neurons to injected depolarizing currents in their presence was undertaken to further establish the concentration-dependent effects of halothane on evoked activity. Depolarizing current pulses of 1-2 nA amplitude and 5 s duration were injected into the soma of neurons. In normal HBS these evoked a response which comprised a discharge of rapidly adapting action potentials in all cells irrespective of whether the cell was normally spontaneously active or silent. Peak to peak amplitude of action potentials was between 80 and 100 mV and frequency between 2 and 4 Hz. In the presence of halothane, however, it soon became apparent that the effect of the anesthetic on evoked activity was concentration–dependent (Figures 9, 10), but also the evoked responses could be characterized on the basis of cell type. The small motor neurons (A group, M group, and J cells) did not generate PDS at 0.5% halothane, but often generated more intense patterned activity (Figure 9A). However, the cells responded with PDS at 1% halothane and became silent at 2.0% halothane, but PDS-like damped action potentials could be evoked by depolarizing stimuli (Figure 9B) whereas this was not the case for the large motor neurons VD1/RPD2, or for the interneurons VV1/2, RPD1, or RPeD1 all of which tended to quiescence and in which no action potentials could be generated.

##### Enflurane

Enflurane had an overall dramatic effect on spontaneous activity of neurons. In 8 different experiments, with concentrations between 0.5 and 4.0% v.v. enflurane, all cells showed a response to the anesthetic within 20 s after the start of superfusion. Unlike results obtained with halothane, neuronal responses to enflurane were not concentration-dependent. Neither the spontaneous activity, nor synaptic patterning showed any trends with concentration. The responses fell into two clear categories in the 8 cells studied. In the presence of enflurane, the spontaneous activity, of 5 of the 8 cells (interneurons RPeD1 x 2, VV1, VV2, and large motor neuron RPD1) became quiescent with no evidence of PDS and (Figure 10A). In the other 3 cells, which were all small motor neurons (A cell, M cell, J cell), in the presence of 1.5-2.0% v.v. enflurane, evoked activity was characterized by the occurrence of PDSs (Figure 10B and spontaneous large amplitude depolarizations ca. 20 mV) were sporadically observed.

##### Isoflurane

The action of isoflurane on spontaneous and evoked neuronal activity was markedly in contrast with those of enflurane (*n* = 57). Within a relatively broad spectrum of concentration (0.5-3.0% v.v.), isoflurane had similar actions at each concentration, i.e., as with enflurane, no concentration-dependence of the neuronal responses was revealed. Furthermore, there was little apparent cell type-dependence in the 6 different cell types examined. In all cells studied isoflurane caused a gradual decline in frequency of spontaneous action potentials during which time, action potential amplitude and AHP amplitude decreased. There was no evidence of PDS-like activity in any of the neurons studied (Figure 10C and see Table 2). Again large amplitude, sporadic depolarizations were observed.

### Comparison of the Electrophysiological Characteristics of Neurons *in situ* and in Culture

#### The Nature of Paroxysmal Depolarizing Shifts

PDS could be generated in a number of different cell types by each of the anesthetics used (Table 2), but it was unclear whether it was synaptically generated or whether it was an endogenous property of the neurons themselves as has been indicated in a preliminary study on the cerebral giant cells of *Lymnaea* (Walcourt-Ambakederemo and Winlow, 1993). Furthermore, it was unclear whether cells identified as exhibiting PDS or tending to quiescence would behave the same way in isolation. We therefore carried out experiments on isolated, identified neurons in culture to determine their action potential characteristics, and their responses to applied halothane.

Here we concentrated on cultured *Lymnaea* interneurons, VV1/2, RPD1, RPeD1, and the motor neurons RPD2 and M group to compare their electrophysiological properties with those *in situ* and to also examine their responses to halothane *in situ* and in culture. The interneurons and giant motor neuron RPD2, are very large in size (100–150 μM in diameter) while the M group neurons form a distinct group with cell bodies about 80 μM in diameter. Hence all were easy to identify and select for cell culture. The electrophysiological activities of these neurons were first recorded in normal saline experiments for each neuron type. The results were then pooled together for the purpose of comparison between the key electrophysiological parameters of resting membrane potential, action potential shape, amplitude and duration *in situ* and in culture. The comparison between these parameters can be seen in Table 3.

**Table 3:** Comparison of the action potential parameters of the different neurons both *in situ* and culture.

	**Action potential shape**		**Action potential amplitude (mV)**		**Action potential half-width (ms) at specified frequency (spikes/s)**	
**Cell type**	***in situ***	**in culture**	***in situ***	**in culture**	***in situ***	**in culture**
M Group Motor neurons	Type 2 *n* = 7	Type 2 (4) *n* = 12	78.42 ± 7.69	80.52 ± 7.37 (NS)	21.10 ± 7.71 Freq ≤ 1.5	16.54 ± 8.19 (S) Freq ≤ 1.5
RPD2 Motor neuron	Type 2 *n* = 8	Type 2 (1) *n* = 8	94.04 ± 5.74	87.10 ± 7.73 (NS)	14.68 ± 2.41 (*n* = 8) Freq = 1.23 ± 0.29	23.37 ± 4.10 (*n* = 8) (S) Freq = 1.20 ± 0.27 (NS)
VV1/2 Putative interneurons	Type 2 *n* = 13	Type 2 (2) *n* = 12	93.79 ± 7.45	85.83 ± 11.94 (S)	8.44 ± 2.59 (*n* = 8) Freq = 0.57 ± 0.16	16.05 ± 4.37 (*n* = 8) (S) Freq = 0.60 ± 0.14 (NS)
RPD1 Interneuron	Type 2 *n* = 13	Type 2 *n* = 14	93.40 ± 7.88	86.89 ± 6.03 (S)	18.25 ± 3.39 (*n* = 8) Freq = 0.85 ± 0.23	22.15 ± 5.03 (*n* = 8)(NS) Freq = 0.82 ± 021 (NS)
RPeD1 Interneuron	Type 2 (1) *n* = 21	Type 2 (3) *n* = 17	95.36± 6.33	81.95 ± 7.32 (S)	7.95 ± 1.65 (*n* = 8) Freq = 1.09 ± 0.30	9.75 ± 1.82 *n* = 8)(NS) Freq = 1.03 ± 0.27 (NS)
Total “n”	62	63				

*This Table compares the action potential (AP) shape, APA, and half-width (HW) at the stated frequencies of examples of motor neurons and interneurons both in situ and in culture. Most neurons retained their AP shapes in culture. The number of times the neurons were found to exhibit type 1 action potentials is given in brackets. The neuron RPeD1 exhibited type 2 and type 1 action potentials both in situ and in culture, whereas, in M group, VV1/2 and RPD2, type 1 action potentials were only expressed in culture, indicating modifications to somatic ion channels. The data are expressed as mean ± S.D. In most cell types APA is significantly (S) less in culture than in situ, with the exception of M group neurons whose amplitudes are not significantly (NS) different from one another. Furthermore M group neurons tend not to fire in culture and were stimulated to fire at frequencies of < 2 spikes/s, exhibiting a reduction in HW in comparison to cells in situ. All other cells exhibited an increase in HW in culture when AP frequency was maintained both in situ and in culture by injecting appropriate current through the stimulating electrode, although this did not reach significance RPD1 or RPeD1. Thus, with the exception of M group, APs in cultured neurons tend to be shorter and broader compared with those recorded in situ. Freq, firing frequency*.

#### Resting Membrane Potential

In the intact brain all the cultured neurons were characterized by a slow regular or irregular pattern of firing whose frequency varied among the neuron types from 0.5 to 1.5 spikes/s Also, in intact preparations, they exhibited a resting membrane potential in the range of −40 to −50 mV. *In situ*, they usually fired spontaneously but sometimes they required the injection of depolarizing current (0.5–1.0 nA) in order to elicit action potentials. In culture, their resting membrane potentials varied between −60 and −80 mV, and hence they were unable to fire unless stimulated with the injection of DC current. All the intracellular recordings made from them in culture were obtained after a continuous injection of a small amount of depolarizing current (0.01–0.06 nA). The only exception to this was in RPD2, where it was found to fire spontaneously in 2 out of 8 experiments (25%) with a relatively lower resting membrane potential (about −40 mV).

#### Action Potential Shape

All this group of neurons exhibited type 2 action potentials *in situ* with the exception of RPeD1, where type 1 action potentials were also observed on some occasions, which is a normal characteristic of this neuron, as mentioned above. In culture, most of the neurons maintained type 2 action potential shapes (53 out of 63, 84.1%) and only a few exhibited type 1 action potentials (10 cells, 15.9%; Table 3).

#### Action Potential Amplitude and Duration

Action potential amplitude (APA) was measured from the peak of depolarization to the peak of the repolarization (i.e., from peak to peak). The action potential duration was determined at half of its amplitude, known as half-width (HW). The method used for the determination of both APA and HW was as previously described (Ahmed et al., 1993). With the exception of M group neurons, Table 3 shows that the action potential amplitudes of all cultured neurons tend to be reduced compared with those *in situ* while the action potential durations were longer compared with those *in situ*. For all the neurons studied, again with the exception of M group cells, there appears to be an inverse relationship between spike height and its duration. The APA of RPeD1 was found to vary between 99.64 ± 5.38 to 82.44 ± 6.46 mV *in situ* and in culture, respectively, which is highly significant at the 1% level (unpaired *t*-test). In the case of HW, both VV1/2 and RPD2 neurons showed a very significant difference between their action potential durations *in situ* and in culture at 1% level.

#### Effects of Halothane on Cultured Neurons

**Figure 11:**
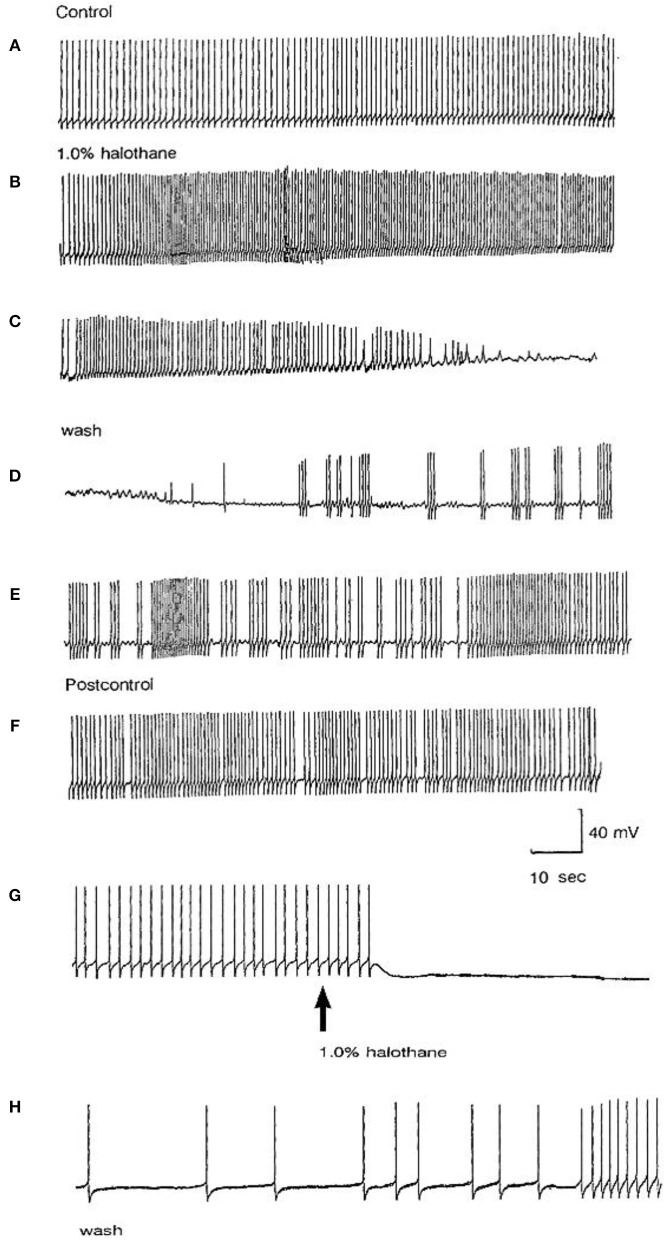
Effect of 1% halothane on two cultured RPeD1 interneurons. The responses of individual cultured neurones are not completely stereotyped as these examples of RPeD1 demonstrate, but all were capable of generating spontaneous action potentials and quickly recovered from the applied anesthetic. **(A)** Under control conditions, the cell was firing regularly **(a)**. Introduction of halothane-containing saline to the chamber led to an increase in the frequency of firing within seconds **(b)**. In less than a minute, the amplitude of the action potential started decreasing along with a diminution of the AHP and depolarization of the cell **(c)**. The cell ultimately stopped firing in a depolarized state, and only slight oscillations could be seen. When the cell was washed with HBS, the membrane potential gradually returned to normal and the oscillations increased **(d)**. Within a couple of minutes, the cell started firing small action potentials which regained their full amplitude in the next 3–4 min **(e)** and the cell returned to almost normal activity in about 5–6 min **(f)**. In another RPeD1 neuron cultured for 2 days, the neuron was firing spontaneously **(g)** until the introduction of halothane, which led to an immediate cessation of spiking activity. The cell hyperpolarized and remained so until washout commenced, during which the cell slowly recovered **(h)** and normal activity resumed in 3–4 min.

The main emphasis of this part of our study was to determine whether isolated neurons could sustain PDS and this turned out to be the case as shown in Table 4. Because most of the cultured cells (92.3%) did not fire spontaneously PDS was usually evoked by intracellular stimulation. Interestingly, PDS could be evoked by 1% halothane in some examples of all the cell types, rather than just M group neurons, as demonstrated in the intact brain, although examples of PDS had sometimes occurred in VV1/2 and RPD1 (Table 2). In addition the responses of individual neurons could be quite variable as shown by the two examples of the neurons RPeD1 in Figure 11.

**Table 4:** Effect of 1.0% halothane on various cell types—M group cells are motor neurons, the other cells are interneurons.

**Cell type**	***n***	**PDS spont**	**PDS evok**	**PDS total**	**Quiescence**
M group	7	0	4	4	3
VV1/2	5	1	3	4	1
RPeD1	10	1	2	3	7
RPD1	4	0	1	1	3
Total “n”	26	2	10	12	14
% of total		7.7	38.4	46.1	53.8

*More than 50% of the 26 cells studied went into quiescence on exposure to halothane. The majority of the cells showing PDS were stimulated by injecting a small amount of current. This current injection could alter the membrane potential and predispose a cell to PDS as certain currents are activated or inactivated at particular membrane potentials. See text for details. key: spon, spontaneous; evok, evoked; PDS, paroxysmal depolarising shifts; n, number of cells studied*.

## Discussion

### Differential Responses of Neurons to Anesthetics

The response of neurons to anesthetics is most often a change in transmembrane voltage and hence in neuronal excitability (Maze, 1990), but as has been demonstrated in this study, these responses are not stereotypical (Tables 1, 2). Results from all the agents used in this report show that specific cells studied in the intact brain have specific responses to anesthetics and their pathways to silence differ profoundly from one cell type to another (Table 1). However, it is clear that some cells exhibit PDS in all the anesthetics tested here, e.g., an A group or M group neurons and J cells, all of which are motor neurons, will exhibit either spontaneous or evoked PDS with the exception of isoflurane in which all neurons become quiescent. The giant interneurons VV1, VV2, RPD1, and RPeD1 tend to quiescence in all the anesthetics tested. It is noteworthy that the strongly electrically coupled neurons motor neurones VD1 and RPD2 fall into both categories, exhibiting PDS in pentobarbital and quiescence in both isoflurane and halothane (see also Qazzaz and Winlow, 2015, 2017). Furthermore, in VD1 and RPD2, both pentobarbital and halothane cause a substantial increase non-dose-dependent E_m_, but apparently by different mechanisms. In VD1/RPD2 the increase in E_m_ is not concentration-dependent in pentobarbital and is accompanied by a non-dose-dependent increase in R_m_ (Qazzaz and Winlow, 2017) indicating a decrease in conductance (g_m_). This is not the case halothane where the large increase in E_m_ is accompanied by a concentration-dependent decrease in R_m_ and thus an increase in gross g_m_ (Figure 6) as previously demonstrated in VV1/VV2 (Winlow et al., 1987) and which also occurs in menthol (Haydon et al., 1982). The underlying mechanisms of these differences remain to be discerned.

### Anesthetics Have Distinctive Effects on Motor Neurons and Interneurons

Many of the neurons we have studied here are involved in the control of the cardiorespiratory system via the respiratory central pattern generator (rCPG) of *Lymnaea* (Syed and Winlow, 1991), which has been reconstructed *in vitro* (Syed et al., 1990). The rCPG also controls cardiac functions (Benjamin and Kemenes, 2013). RPeD1 projects directly to the osphradial ganglion and receives direct excitatory, monosynaptic, cholinergic inputs from osphradial neurons (Bell et al., 2007) which are responsive to hypoxia (Janes and Syed, 2012). The parietal A group neurons innervate the musculature of the mantle cavity, while the visceral J cells are pneumostome opener motor neurones and some pneumostome closer motor neurons are thought to be located within visceral M group (Moroz, 1991). The strongly electrically coupled neurons VD1 and RPD2 are hypoxia and osmo-sensitive, peptidergic motor neurons which directly innervate the auricle (Kerkhoven et al., 1991) and are thought to modulate heart rate (Benjamin and Kemenes, 2013), but which also project to the skin near the pneumostome, osphradium, and lips. They are unlikely to have a direct sensory function since they were not labeled after back filling the osphradial nerve with either Ni-lysine or biocytin (Nezlin, 1995). The giant neurons VV1 and VV2 are responsive to osmotic stimulation of the osphradium (Kamardin, 1995), but are unlikely to be primary sensory neurones as they did not backfill from the osphradial nerve (Nezlin, 1995). They currently have no known motor function. Finally, the neuron RPD1 is in all likelihood an interneuron which branches into peripheral nerves and which receives polymodal sensory inputs from the skin of the tentacles lips and mantle (Zaitsev and Shuvalova, 1988) including inhibitory non-ocular photo-responses from the foot through the pedal nerves to the pedal ganglia (Chono et al., 1992).

#### Motor Neurons Exhibit PDS, but Interneurons Do Not Do So in the Intact Brain

A comparison of the responses of these different neuron types to applied anesthetics *in situ* indicate that, except in isoflurane, the four types of motor neurons studied here, A group, J cells, M group VD1/RPD2, are all capable of generating PDS while the interneurons RPeD1 (Syed et al., 1991) and RPD1 as well as the presumed interneurons VV1/VV2 all tend to quiescence in the presence of applied anesthetics in the intact brain (Table 2). Similar effects have also been observed in 1% halothane in the paired buccal 3-cells (McCrohan et al., 1987; Girdlestone et al., 1989b) and in cultured 4-cells (Walcourt and Winlow, both of which are motor neurons. Thus, the motor neurons and interneurons discussed here may be distinguished by their responses to anesthetics, but further work will be required to see if this hypothesis can be generalized, particularly as it is unclear whether other buccal interneurons exhibit PDS. The reasons for the differences in responses are not simply due to cell size (see Figure 1) and in isolated cell culture the interneurons were more capable of generating PDS than in the intact brain (Table 4), suggesting that PDS may be an endogenous property of all types of neurons (see below), but modified by their interactions with other cells, in terms of the channels expressed by individual neurons or activated by neurotransmitters, either in the intact nervous system or when grown in culture with synaptically connected cells. Interestingly menthol has effects on motor neurons and interneurons (Haydon et al., 1982) similar to those described here. Menthol is now known to block voltage-dependent sodium channels in rat neurons and human skeletal muscle and shares analgesic (Galeotti et al., 2002) and anesthetic properties with propofol (Watt et al., 2008) by its modulatory action on GABA_A_ receptors (Lau et al., 2014). On the basis of this information it is conceivable that PDS may have a role in the early excitatory phase of anesthesia when uncoordinated movements in *Lymnaea* and other animals can be observed in the absence of sedation or neuromuscular blocking agents.

### Effects of Anesthetics on Membrane Potential and Spontaneous Activity

Apart from VD1/RPD2, pentobarbital, and halothane at all concentrations used in the present study caused membrane hyperpolarization, usually of only a few millivolts (Table 1), except in M group neurons (Figure 3A) which depolarized in pentobarbital. The reasons for this unexplained depolarization remain to be investigated using appropriate patch clamp methodology. Nicoll and Madison (Nicoll and Madison, 1982) have reported that general anesthetics hyperpolarize neurons in the vertebrate central nervous system, due to an increase in potassium, and not chloride, permeability. O'Beirne et al. (1987) have reported neuronal hyperpolarization, decreased spontaneous activity, and sometimes decreased input resistance after administration of different concentrations of pentobarbital in albino guinea pig hippocampal slices. They proposed that pentobarbital caused neuronal inhibition, particularly at low doses, due to an increase in potassium conductance (O'Beirne et al., 1987). Blaustein (1968) has demonstrated that pentobarbital and thiopentone blocked sodium and potassium conductances under voltage clamp in the lobster. The issue of how barbiturates and especially pentobarbital affect potassium currents, thus remains controversial. We have seen partial blockage of gross K^+^ currents (Moghadam and Winlow, 1995; Moghadam, 1996) when different concentrations of pentobarbital are applied to the neurons. The differences in outcome may arise from different preparations and different methods employed, but may also be due to different K^+^ channels responding in different ways. In addition, halothane has been shown to block L-type calcium current in cultured *Lymnaea* neurons in a concentration-dependent manner (Yar and Winlow, 2016b), but of course the precise mix of calcium and potassium channels is likely to vary from one neuron type to another.

### Effects of Anesthetics on the After Hyperpolarization and Pseudoplateau

The AHP, which is a calcium-dependent phenomenon, was reversibly reduced or abolished by pentobarbital in all cell types except RPeD1 (see Table 1) and was markedly reduced in halothane (Figures 7–9). Reversibility was clearer and more rapid in halothane than in pentobarbital. Three different AHPs with different time courses exist in hippocampal neurons which are sensitive to the volatile anesthetic isoflurane and it has been reported that isoflurane decreased the AHP in rat hippocampal and human neocortical neurons (Berg-Johnsen and Langmoen, 1990). The AHP, which occurs after an action potential and is due to a Ca^2+^ activated K^+^ conductance (Hoston and Prince, 1980; Schwartz-Kroin and Stafstrom, 1980) decreased significantly in the PDS group of neurons in response to pentobarbital applications (Figures 2, 3) and it was slow to recover after washout of the drug (Figure 3Aa) even though the membrane potential (E_m_) had recovered. This phenomenon suggests that the response of components of the AHP in these neurons is time dependent with respect to pentobarbital application. Different form of PDS Figures 3Ag,Bb doublet spiking) compared with earlier or later responses in the same neurons, which suggests that different ionic channels with different kinetics and time-dependencies may be involved in the generation of the different types of PDS.

*Lymnaea* neurons generate two different types of action potentials categorized as type 1 (typical neuronal action potential) and type 2 (action potential with a calcium-dependent pseudoplateau) (Gardner and Kerkut, 1968; Winlow and Benjamin, 1976; Girdlestone, 1986). The action potential type of RPeD1 is variable (Kyriakides et al., 1989), but is most usually type 2 and this variability may be due to seasonal factors (Wood and Winlow, 1996; Copping et al., 2000). The influx of Ca^2+^ during action potentials is supposed to be sufficient to activate Ca^2+^ dependent potassium channels (Meech and Standen, 1974). We have previously demonstrated that pentobarbital (1 and 2 mM) reversibly decreased action potential half width (Moghadam and Winlow, 1993), which is a good measure of the reduction of the width of the pseudoplateau in type 2 molluscan action potentials. This plateau is generated by an L-type calcium current (Yar and Winlow, 2016a) and pentobarbital can also reduce L and N (not T) type Ca^2+^ currents in cultured, vertebrate, sensory neurons in a dose dependent manner (Gross and Macdonald, 1988). The effects on discharge would be to reduce frequency, spike width and AHP of the action potential. This is similar to previous findings, which have shown that the calcium components of action potentials are decreased by barbiturates (Morgan and Bryant, 1977; Heyer and Macdonald, 1982). Pentobarbital (at 25–600 mM) and phenobarbitone (100–5,000 mM) reduce action potential duration with sedative and anesthetic doses in large multipolar spinal cord neurons (Heyer and Macdonald, 1982). In contrast, in the M group neurons we have found pentobarbital at low doses increases activity (Figure 3) as previously demonstrated by Larrabee and Posternak (1952) who reported that pentobarbital enhances transmission at lower concentrations.

#### Concentration Dependence of Anesthetics

There is no obvious trend between volatile anesthetic concentrations and their effects at a cellular level with the two inhalation anesthetics, enflurane, and isoflurane, which is in sharp contrast to results obtained with halothane. Despite the use of a relatively wide concentration range, i.e., 0.25–4.00%, there was little difference in the response produced by each. Both the anesthetics did however, have immediately apparent effects on neuronal activity. Enflurane caused various dramatic changes in neuronal behavior very soon after superfusion, which are discussed in more detail below, and isoflurane caused immediate quiescence irrespective of the concentration. The absence of a concentration dependent effect with these two agents at a neuronal level is difficult to explain. However, in these experiments concentration dependence was judged by observing concentration related changes in spontaneous and evoked neuronal activity. It is possible that concentration dependence is not revealed at this level, and because these two agents are less prone than halothane to cause an excitatory phase. However, concentration dependent changes have been noted in terms of conductance changes, induced by halothane and isoflurane in VD1/RPD2 (Qazzaz and Winlow, 2015, 2017) and patch clamp studies reveal that both calcium currents (Yar and Winlow, 2016b) and potassium currents (Winlow et al., 1995; Moghadam, 1996) are depressed in a concentration-dependent manner by halothane in cultured, identified neurons.

### Is PDS an Endogenous or a Synaptically Driven Phenomenon?

PDS-like activity can be triggered synaptically in *Lymnaea* by the action of the input 3 interneuron on the J cell motor neurons (Benjamin and Winlow, 1981) and has been described elsewhere, especially in relation to epilepsy (Prince, 1968, 1978; Speckmann and Caspers, 1973; Lux, 1984; Jefferys, 2010). PDS has been investigated to determine whether it is an endogenous or synaptically mediated phenomenon (Speckmann and Caspers, 1973). Epilepsy is probably driven collectively by large numbers of interconnected neurons, many with intrinsic membrane properties that allow the emergence of PDS (Jefferys, 2010). A PDS, which leads to damped burst of spikes, is a large Pathak (2017), abnormal Kubista et al. (2019) depolarizing wave caused by suppression of calcium-activated potassium currents due to blockade of voltage-gated potassium currents that then unmask persistent sodium currents (Pathak, 2017) Such calcium-activated potassium currents are found in *Lymnaea* neurons (Moghadam, 1996).

There is a wide range of potassium currents in *Lymnaea* neurons (see Winlow et al., 2018 for review) and preliminary papers have demonstrated that halothane, isoflurane and pentobarbital depress gross potassium currents in a dose-dependent manner (Moghadam and Winlow, 1995; Winlow et al., 1995; Moghadam, 1996). In addition, potassium channel blockers such as tetraethylammonium and 4-aminopyridine have been shown to induce PDS in *Lymnaea* neurons (Holden et al., 1982b, 1983) including doublet and triplet spiking (Holden and Winlow, 1982, 1983; Holden et al., 1982a, 1983) similar to that seen in Figure 3Bb. Zero calcium saline can also have the same effect (Holden et al., 1983). It should be noted that although PDS does not normally occur in the cerebral giant cells (CGCs), which are interneurons innervating the buccal ganglia, either in the whole brain or in culture, it can be elicited in exceptional, experimental circumstances when cultured CGCs are subjected not only to 1% halothane, but to 1% halothane in zero Ca2+/high Mg2+/1 mM EGTA saline (Walcourt-Ambakederemo and Winlow, 1993), supporting the view that PDS may be an endogenous property of most neurons, given the right conditions. Calcium currents are involved in the pseudoplateau and the AHP of *Lymnaea* neurons as mentioned above. They have recently been characterized as high voltage activated L-type calcium currents in pedal I cluster neurons (Yar and Winlow, 2016a), which have type 2 action potentials. Their calcium currents are suppressed in a concentration-dependent manner by halothane (Yar and Winlow, 2016b). Thus, there is a panoply of potassium and calcium channels likely to be involved in the generation of PDS within individual *Lymnaea* neurons [for more detail, see (Winlow et al., 2018)]. Within the vertebrate nervous system, both synaptic (Cisse et al., 2004) and endogenous membrane currents are thought to be involved in the generation of PDS (Feher et al., 1982). A further suggestion is that PDS may be due to Cl^−^ -dependent depolarizing postsynaptic potentials in pyramidal neurons (Timofeev et al., 2002a,b). Whether both mechanisms can occur in the same neuron remains to be seen. Finally several authors suggest that perturbations of intracellular calcium concentration [Ca]_i_ may be associated with PDS (Raza et al., 2004; Kubista et al., 2019) and that raised [Ca]_i_ is associated with acquired epilepsy in hippocampal neurons. Both halothane and pentobarbitone are known to raise [Ca]_i_ in *Lymnaea* neurons (Winlow et al., 2018) in common with vertebrate neurons (Mody et al., 1991). Thus, the rise in [Ca]_i_ may be sufficient to trigger PDS in susceptible cells even though halothane is known to block L-type calcium currents (Yar and Winlow, 2016b).

Here, we have demonstrated that PDS can be generated by halothane in isolated, cultured neurons and this finding is supported in a preliminary study on the cerebral giant cells of *Lymnaea* (Walcourt-Ambakederemo and Winlow, 1993). In the cultured neurons described in the current report, PDS can sometimes be evoked in all the cell types studied (Table 4), particularly in RPeD1 in which PDS has not been observed in the intact brain (Table 2). This implies that the neurons are plastic and that machinery to generate PDS may be present in all neuron types, but that the membrane currents generating it are modulated depending on the synaptic and network milieu in which an individual cell finds itself *in vivo*. Given that the cultured *Lymnaea* neurons retain their basic action potential shapes (Table 3 and see Winlow et al., 1991, 1992, 2018; Yar and Winlow, 1991), their transmitter identity (Syed et al., 1990; Spencer et al., 1995; Naruo et al., 2005), their responsiveness to applied transmitters Haydon, 1989; Syed et al., 1990 and can be reconstructed into meaningful circuits *in vitro* Syed et al., 1990, they are powerful tools for cellular studies of the action of anesthetic agents. It is of interest that the dampened action potential bursts, characteristic of PDS were never observed in isoflurane, but spontaneous and evoked large depolarizing potentials still occurred in its presence in RPD1 (Figure 10Cb) which may be the source of PDS, but this remains to be elucidated. However, similar depolarizations are also found in M group cells in enflurane where evoked PDS could be generated (Figures 10Bbc) and similar depolarizing events were also noted in VV1/VV2 in enflurane. Such events are unlikely to be synaptic in origin as they still occur at high concentration of enflurane (3%−21) which would be expected to cause complete anesthesia in most animals and would be expected to block synaptic transmission between cultured *Lymnaea* neurons (Spencer et al., 1995, 1996). Thus, the depressant effects of anesthetics on calcium and potassium currents, accompanied by rising [Ca^2+^]_i_, may well trigger the depolarizing wave underlying PDS in many types of neuron, although this is often not observed due to suppression of action potentials in these cells.

### Relevance of Anesthetic Studies on *Lymnaea* to Studies of Anesthetic Actions on Cephalopod Molluscs

The Cephalopod mollusc *Octopus vulgaris* is now classed as a sentient being (UK Statutory Instruments, 1993; European Parliament European Union, 2010) and should be treated as such when it is used for experimental purposes in the same way as vertebrates so as to reduce pain and suffering. A recent report (Polese et al., 2014), based on previous studies on *Lymnaea* (Girdlestone et al., 1989a,c) has shown that *Octopuses* can be anesthetized with isoflurane and recover well from this procedure. Further studies are now required to refine these procedures. Given that gastropod molluscs are evolutionarily closer relatives to cephalopods than are vertebrates, it is probable that the responsiveness of gastropods to anesthetic agents will yield clues for the future development of cephalopod anesthesia.

## Conclusion

Neuronal responses to anesthetics are not stereotyped and are characteristic of motor neurons and interneurons in *Lymnaea stagnalis in situ*. The small motor neurons studied here typically generated paroxysmal depolarizing shifts during the application of halothane, enflurane, pentobarbital, thiopentone, and ketamine, but the giant motor neurones only did so in pentobarbital. The interneurons did not usually generate PDS. No cells exhibited PDS in isoflurane and all became quiescent. Examples of motor neurons and interneurons, when isolated in short term culture, were shown to be capable of generating PDS.

## Ethics Statement

This work was carried out on specimens of the pond-snail *Lymnaea stagnalis* which are exempt from this type of approval, but which were maintained in humane conditions in any case.

## Author Contributions

The basic concept behind this work was generated by WW in whose laboratory (originally in the Department of Physiology, University of Leeds, UK) all the co-authors worked as graduate students completing their PhDs in a timely manner. WW constructed the present paper from their combined their data. All the co-authors contributed equally.

### Conflict of Interest Statement

The authors declare that the research was conducted in the absence of any commercial or financial relationships that could be construed as a potential conflict of interest.

## References

[B1] Ahmed I. A. Hopkins P. M. Winlow W. (1993). Caffeine and ryanodine modify the half-width of somatic action potentials in identified molluscan neurones. J. Physiol. 459:13.

[B2] Ahmed I. A. Hopkins P. M. Winlow W. (1997). Low concentrations of caffeine raise intracellular calcium concentration only in the presence of extracellular calcium in cultured molluscan neurones. Gen. Pharmac. 28:245–250. 10.1016/S0306-3623(96)00233-9 9013202

[B3] Bell H. J. Inoue T. Shum K. Luk C. Syed N. I. (2007). Peripheral oxygen sensing cells directly modulate the output of an identified respiratory central pattern generating neuron. Eur. J. Neurosci. 25:3537. 10.1111/j.1460-9568.2007.05607.x 17610573

[B4] Benjamin P. R. Kemenes G. (2013). *Lymnaea* neuropeptide genes. Scholarpedia 8:11520 10.4249/scholarpedia.11520

[B5] Benjamin P. R. Winlow W. (1981). The distribution of wide-acting Synaptic input to identified neurons in the isolated brain of *Lymnaea stagnalis* (L.). Comp. Biochem. Physiol. 70A, 293–307. 10.1016/0300-9629(81)90182-1

[B6] Berg-Johnsen J. Langmoen I. A. (1990). Mechanisms concerned in the direct effect of isoflurane on rat hippocampal and human neocortical neurons. Brain Res. 507, 28–34. 10.1016/0006-8993(90)90517-F 2302577

[B7] Blaustein M. P. (1968). Barbiturates block sodium and potassium conductance increases in voltage-clamped lobster axons. J. Gen. Physiol. 51, 293–307. 5648829 10.1085/jgp.51.3.293 PMC2201133

[B8] Bowman W. C. Rand M. J. (1980). Textbook of Pharmacology. 2nd ed. Oxford: Blackwell scientific Publications.

[B9] Chono K. Fujito Y. Ito E. (1992). Non-ocular dermal photoreception in the pond snail *Lymnaea stagnalis*. Brain Res. 951, 102–112. 10.1016/S0006-8993(02)03143-8 12231463

[B10] Cisse Y. Crochet S. Timofeev I. Steriade M. (2004). Synaptic responsiveness of neocortical neurons to callosal volleys during paroxysmal depolarising shifts. Neuroscience 124, 231–239. 10.1016/j.neuroscience.2003.11.003 14960354

[B11] Copping J. Syed N. I. Winlow W. (2000). Seasonal plasticity of synaptic connections between identified neurones in *Lymnaea*, in Neurobiology of Invertebrates. Membranes, Chemical Signalling and Systems Approach, eds Elekes K. Salanki J. (Budapest: Akademia Kiado), 205–210. 11034145

[B12] European Parliament and European Union (2010). Council on the protection of animals used for scientific purposes, in Directive 2010/63/EU No. L276, ed European Union (Croatia: Official Journal of the European Union), 33–79.

[B13] Feher O. Baranyi A. Gyimothy T. (1982). A model for the ionic mechanism of the paroxysmal depolarisation shift, in Physiology and Pharmacology of Epileptogenic Phenomena, eds lee M. R.K (New York, NY: Raven Press). p. 343–351.

[B14] Franks N. P. Lieb W. R. (1988). Volatile general anaesthetics activate a novel neuronal K^+^ current. Nature 333, 662–664. 10.1038/333662a0 2453807

[B15] Galeotti N. Di Cesare Mannelli L. Di C. Mazzanti G. Bartolini A. Ghelardini C. (2002). Menthol: a natural analgesic compound. Neurosci. Lett. 322, 145–148. 10.1016/S0304-3940(01)02527-7 11897159

[B16] Gardner D. R. Kerkut G. A. (1968). A comparison of the effects of sodium and lithium ions on action potentials from *Helix aspersa* neurons. Comp. Biochem. Physiol. 25, 33-48. 10.1016/0010-406X(68)90912-2 5657210

[B17] Girdlestone D. (1986). Electrophysiological Studies of the Actions of General Anaesthetics on Identified Molluscan Neurones and Neuronal Networks. (PhD Thesis), University of Leeds.

[B18] Girdlestone D. Cruickshank S. G. H. Winlow W. (1989a). The actions of three volatile general anaesthetics on the withdrawal reflexes of *Lymnaea stagnalis* (L). Comp. Biochem. Physiol. 92C, 39–43. 10.1016/0742-8413(89)90199-0 2566441

[B19] Girdlestone D. Cruickshank S. G. H. Winlow W. (1989c). A system for the application of general anaesthetics and other volatile agents to superfused isolated tissue preparations. Comp. Biochem. Physiol. 92C, 35–37. 10.1016/0742-8413(89)90198-9 2566440

[B20] Girdlestone D. McCrohan C. R. Winlow W. (1989b). The actions of halothane on spontaneous activity, action potential shape and synaptic connections of the giant serotonin-containing neurones of *Lymnaea stagnalis* (L). Comp. Biochem. Physiol. 93C, 333–339. 10.1016/0742-8413(89)90243-0 2572388

[B21] Gross R. A. Macdonald R. (1988). Differential actions of pentobarbital on calcium current component of sensory neurons in culture. J. Physiol. 405, 187–203. 10.1113/jphysiol.1988.sp017328 2855640 PMC1190971

[B22] Guedel A. E. (1937). Inhalational Anesthesia; A Fundamental Guide. New York, NY: Macmillan.

[B23] Haydon P. G. (1989). Formation of chemical synapses: neuronal strategies, in The Cellular Basis of Neuronal Plasticity – Physiology, Morphology and Biochemistry of Molluscan Neurons Studies in Neuroscience, ed Bulloch A. G. M. (Manchester: Manchester University Press), 129–151.

[B24] Haydon P. G. Winlow W. Holden A. V. (1982). The effects of menthol on central neurons of the pond-snail, *Lymnaea stagnalis* (L). Comp. Biochem. Physiol. 73C, 95–100. 10.1016/0306-4492(82)90174-5

[B25] Heyer E. J. Macdonald R. L. (1982). Barbiturate reduction of calcium-dependent action potentials: correlation with anesthetic action. Brain Res. 236, 157–171. 10.1016/0006-8993(82)90042-7 6279233

[B26] Holden A. V. Haydon P. G. Winlow W. (1983). Multiple equilibria and exotic behaviour in excitable membranes. Biol. Cyber. 46, 167–172. 10.1007/BF00336798 6850003

[B27] Holden A. V. Winlow W. (1982). Bifurcation of periodic activity from periodic activity in a molluscan neurone. Biol. Cyber. 42, 189–194. 10.1007/BF00319976 7093359

[B28] Holden A. V. Winlow W. (1983). Neuronal activity as the behaviour of a differential system. IEEE Trans. Syst. Man. Cybernet Special Issue 13, 711–719. 10.1109/TSMC.1983.6313064

[B29] Holden A. V. Winlow W. Haydon P. G. (1982a). The induction of periodic and chaotic activity in a molluscan neuron. Biol. Cyber. 43, 169–173. 10.1007/BF00319976 7093359

[B30] Holden A. V. Winlow W. Haydon P. G. (1982b). Effects of tetraethylammonium and 4-aminopyridine on the somatic potentials of an identified molluscan neuron. *Comp*. Biochem. Physiol. A 73A, 303–310. 10.1016/0300-9629(82)90075-5 6128118

[B31] Hoston J. R. Prince D. A. (1980). A calcium-activated hyperpolarisation follows repetitive firing in hippocampal neurons. J. Neurophysiol. 43, 409–419. 10.1152/jn.1980.43.2.409 6247461

[B32] Janes T. A. Syed N. I. (2012). Neuronal mechanisms of oxygen chemoreception: an invertebrate perspective, in Arterial Chemoreception, eds Nurse C. Gonzalez C. Peers C. Prbhakar N. (Dordrecht: Springer), 7–17. 10.1007/978-94-007-4584-1_2 23080137

[B33] Jefferys J. G. R. (2010). Advances in understanding basic mechanisms of epilepsy and seizures. Seizure 19, 638–646. 10.1016/j.seizure.2010.10.026 21095139

[B34] Kamardin N. N. (1995). The electrical responses of osphradial nerve and central neurons to chemical stimulation of *Lymnaea* osphradium. Acta Biol. Hungar. 46, 315–320. 8853702

[B35] Kerkhoven R. M. Croll R. P. Van Minnen J. Bogerd J. Ramkema M. D. Lodder H. (1991). Axonal mapping of the giant peptidergic neurons VD1 and RPD2 loacted ih the CNS of the pond snail *Lymnaea stagnalis*, with particular reference to the auricle of the heart. Brain Res. 565, 8–16. 10.1016/0006-8993(91)91730-O 1723025

[B36] Kubista H. Boehm S. Hotka M. (2019). The paroxysmal depolarization shift: reconsidering its role in epilepsy, epileptogenesis and beyond. Int. J. Mol. Sci. 20:577. 10.3390/ijms20030577 30699993 PMC6387313

[B37] Kyriakides M. McCrohan C. R. Slade C. T. Syed N. I. Winlow W. (1989). The morphology and electrophysiology of the neurones of the paired pedal ganglia of *Lymnaea stagnalis* (L). Comp. Biochem. Physiol. 93A, 861–887. 10.1016/0300-9629(89)90513-6 2570671

[B38] Larrabee M. G. Posternak J. M. (1952). Selective action of anesthetics on synapses and axon in mammalian synaptic ganglia. J. Neurophysiol. 15, 91–141. 10.1152/jn.1952.15.2.91 14908628

[B39] Lau B. K. Karim S. Godchild A. K. Vaughan C. W. Drew G. M. (2014). Menthol enhances phasic and tonic GABAA receptor-mediated currents in midbrain periaqueductal grey neurons. Br. J. Pharmacol. 171, 2803–2813. 10.1111/bph.12602 24460753 PMC4243856

[B40] Lux D. (1984). An invertebrate model of paroxysmal depolarizing shifts, in Electrophysiology of Epliepsy, eds Schwartzkroin P. A. Wheal H. V. (London: Academic Press).

[B41] Maze M. (1990). Transmembrane signalling and the holy grail of anesthesia. Anesthesiology. 72, 959–961. 10.1097/00000542-199006000-00001 2161622

[B42] McCrohan C. R. Girdlestone D. Winlow W. (1987). Effects of halothane on feeding motor activity in *Lymnaea stagnalis*. Comp. Biochem. Physiol. 86C, 55–62. 10.1016/0742-8413(87)90144-7 2881727

[B43] McKenzie D. Franks N. P. Lieb W. R. (1995). Actions of general anaesthetics on a neuronal nicotinic acetylcholine receptor in isolated identified neurones of *Lymnaea stagnalis*. Br. J. Pharmacol. 115, 275–282. 10.1111/j.1476-5381.1995.tb15874.x 7670729 PMC1908331

[B44] Meech R. W. Standen N. B. (1974). Potassium activation in *helix* aspersa neurons under voltage clamp:a component mediated by calcium. J. Physiol. 249, 211–230. 10.1113/jphysiol.1975.sp011012 PMC1309571 1177091

[B45] Mody L. Tanelian D. L. MacIver M. B. (1991). Halothane enhances tonic neuronal inhibition by elevating intracellular calcium. Brain Res. 538, 319–323. 10.1016/0006-8993(91)90447-4 1901506

[B46] Moghadam H. F. (1996). Effects of General Anesthetics and Free Radicals on Cultured Identified Neurons of Lymnaea stagnalis. (Ph.D Thesis), University of Leeds.

[B47] Moghadam H. F. Winlow W. (1993). Pentobarbital modifies action potentials of molluscan neurons, in XXXll Congress of the International Union of Physiological Sciences, Glasgow, 42.

[B48] Moghadam H. F. Winlow W. (1995). Pentobarbital inhibits K^+^ currents in cultured neurons. J. Physiol. 483:191.

[B49] Morgan K. A. Bryant S. H. (1977). Pentobarbital: presynaptic effect in squid giant synapse. Experientia 33, 487–488. 10.1007/BF01922226 862741

[B50] Moroz L. L. (1991). Monoaminergic control of respiratory behaviour in the freshwater pulmonate snail, *Lymnaea stagnalis (L.)*, in Signal Molecules and Behaviour, eds Winlow W. Vinogradova O. S. Sakharov D. A. (Manchester: Manchester University Press), 101–23.

[B51] Naruo H. Onizuka S. Prince D. Takasaki M. Syed N. I. (2005). Sevoflurane blocks cholinergic synaptic transmission postsynaptically but does not affect short-term potentiation. Anesthesiology 102, 920–928. 10.1097/00000542-200505000-00010 15851878

[B52] Nezlin L. P. (1995). Primary sensory neurons and their central projections in the pond snail *Lymnaea stagnalis*. Acta Biol. Hungar. 46, 305–313. 8853701

[B53] Nicoll D. A. Madison D. V. (1982). General anesthetics hyperpolarize neurons in the vertebrate central nervous system. Science. 217, 1055–1056. 10.1126/science.7112112 7112112

[B54] O'Beirne M. Gurevich N. Carlen P. L. (1987). Pentobarbital inhibits hippocampal neurons by increasing potassium conductance. Can. J. Physiol. Pharmacol. 65, 36–41. 10.1139/y87-007 3567717

[B55] Pathak D. (2017). Paroxysmal depolarization shift in leech Retzius nerve cells revisited. MOJ Anat Physiol. 3, 7–9. 10.15406/mojap.2017.03.00077

[B56] Polese G. Winlow W. Di Cosmo A. (2014). Dose-dependent effects of the clinical aesthetic isoflurane on *Octopus vulgaris*: a contribution to cephalopod welfare. J. Aquat. Health 26, 285–294. 10.1080/08997659.2014.945047 25369208

[B57] Prince D. A. (1968). The depolarization shift in epileptic neurons. Exp. Neurol. 21, 467–485. 10.1016/0014-4886(68)90066-6 5677264

[B58] Prince D. A. (1978). Neurophysiology of epilepsy. Ann. Rev. Neurosci. 1, 395–415. 10.1146/annurev.ne.01.030178.002143 386906

[B59] Qazzaz M. M. Winlow W. (2015). Differential actions of volatile anaesthetics and a systemic barbiturate on strongly electrically coupled neurons. EC Neurol. 2, 188–204.

[B60] Qazzaz M. M. Winlow W. (2017). Modulation of the passive membrane properties of a pair of strongly electrically coupled neurons by anaesthetics. EC Neurol. 6, 187–200.

[B61] Rang H. P. Dale M. M. Ritter J. M. Flower R. J. (2007). Rang and Dale's Pharmacology – 6th ed. Churchill Livingstone, PA: Elsevier, 829.

[B62] Raza M. Blair R. E. Sombati S. Carter D. S. Deshpande L. S. DeLorenzo R. J. (2004). Evidence that injury-induced changes in hippocampal neuronal calcium dynamics during eplileptogenesis cause acquired epliepsy. Proc. Natl. Acad. Sci. U.S.A. 101, 17522–17527. 10.1073/0408155101 15583136 PMC535000

[B63] Richards C. D. (1983). Actions of general anesthetics on synaptic transmission in the CNS. Br. J. Anesth. 55, 201–207. 10.1093/bja/55.3.201 6131686

[B64] Schwartz-Kroin P. A. Stafstrom C. E. (1980). Effects of EGTA on the calcium-activated afterhyperpolarisation in hippocampal CA3 pyramidal cells. Science 210, 1125–1126. 10.1126/science.6777871 6777871

[B65] Slade C. T. Mills J. Winlow W. (1981). The neuronal organisation of the paired pedal ganglia of *Lymnaea stagnalis (L)*. Comp. Biochem. Physiol. 69A, 789–803. 10.1016/0300-9629(89)90513-6 2570671

[B66] Speckmann E. J. Caspers H. (1973). Paroxysmal depolarisation and changes in action potentials induced by pentylenetetrazol in isolated neurons of Helix pomatia. Epilepsia 14, 397–408. 10.1111/j.1528-1157.1973.tb03979.x 4521096

[B67] Spencer G. E. Syed N. I. Lukowiak K. Winlow W. (1995). Halothane-induced synaptic depression at both *in vivo* and *in vitro* reconstructed synapses between identified *Lymnaea* neurons. J. Neurophysiol. 74, 2604–2613. 10.1152/jn.1995.74.6.2604 8747218

[B68] Spencer G. E. Syed N. I. Lukowiak K. Winlow W. (1996). Halothane affects both inhibitory and excitatory synaptic transmission at a single identified molluscan synapse, *in vivo* and *in vitro*. Brain Res. 714, 38–48. 10.1016/0006-8993(95)01450-0 8861607

[B69] Syed N. I. Bulloch A. G. Lukowiak K. (1990). *In vitro* reconstruction of the respiratory central pattern generator of the mollusk *Lymnaea*. Science 250, 282–285. 10.1126/science.2218532 2218532

[B70] Syed N. I. Harrison D. Winlow W. (1991). Respiratory behaviour in the pond snail *Lymnaea stagnalis*. I Behavioral analysis and the identification of motor neurons. J. Comp. Physiol. A 169, 541–555. 10.1007/BF00193545

[B71] Syed N. I. Winlow W. (1991). Respiratory behaviour in the pond snail *Lymnaea stagnalis*. II Neural elements of the central pattern generator. J. Comp. Physiol. A 169, 557–568. 10.1007/BF00193546

[B72] Timofeev I. Bazhenov M. Sejnowski T. Steriade M. (2002b). Cortical hyperpolarisation-activated depolarising current takes part in the generation of focal paroxysmal activities. Proc. Natl. Acad. Sci. U.S.A. 9:9533–9537. 10.1073/pnas.132259899 PMC123175 12089324

[B73] Timofeev I. Grenier F. Steriade M. (2002a). The role of chloride-dependent inhibition and the activity of fast-spiking neurons during cortical spike-wave electrographic seizures. Neuroscience. 114, 1115–1132. 10.1016/S0306-4522(02)00300-7 12379264

[B74] UK Statutory Instruments (1993). The Animals (Scientific Procedures) Act (Amendment) Order 1993 Number 2103. London: UK Government.

[B75] Walcourt A. Winlow W. (2019). A comparison of the electrophysiological characteristics of identified neurons of the feeding system of Lymnaea stagnalis (L.) *in situ* and in culture. EC Neurol. 11, 323–333.

[B76] Walcourt-Ambakederemo A. Winlow W. (1994). 5-HT receptors on identified *Lymnaea* neurones in culture. Pharmacological characterization of 5-HT_1A_ receptors. Comp. Biochem. Physiol. 107C, 129–141. 10.1016/1367-8280(94)90019-1 7875529

[B77] Walcourt-Ambakederemo A. W. Winlow W. (1993). Halothane induces paroxysmal depolarisation shifts in synaptically isolated *Lymnaea* neurons in culture. J. Physiol. 473:187.

[B78] Watt E. E. Betts B. A. Kotey F. O. Humbert D. J. Griffith T. N. Kelly E. W.. (2008). Menthol shares general anesthetic activity and sites of action on the GABA(A) receptor with the intravenous agent, propofol. Eur. J. Pharmacol. 590, 120–126. 10.1016/j.ejphar.2008.06.003 18593637

[B79] Winlow W. Benjamin P. R. (1976). Neuronal mapping of the brain of the pond-snail, *Lymnaea stagnalis* (L), in Neurobiology of Invertebrates, Gastropoda Brain, ed Salanki J. (Budapest: Akademiai Kiado), 41–59.

[B80] Winlow W. Girdlestone D. Cruickshank S. G. H. McCrohan C. R. (1987). Lymnaea in the arms of morpheus, in Neurobiology, Molluscan Models, eds Boer H. H. Geraerts W. P. M. Joose J. (Amsterdam: Wetensch North Holland Publ. Co), 132–137.

[B81] Winlow W. Haydon P. G. Benjamin P. R. (1981). Multiple postsynaptic action of the giant dopamine-containing neuron RPeD1 of *Lymnaea stagnalis*. J. Exp. Biol. 4, 137–148.

[B82] Winlow W. Hopkins P. M. Moghadam H. F. Ahmed I. A. Yar T. (1995). Multiple cellular and subcellular actions of general anesthetics on cultured molluscan neurons. Acta Biol. Hungar. 46, 381–393. 8853709

[B83] Winlow W. Polese G. (2014). A neuroplastic network underlying behavior and seasonal change in *Lymnaea stagnalis*: a neuroecological standpoint, in Neuroecology and Neuroethology in Molluscs: The Interface Between Behavior and Environment, eds Di Cosmo A. Winlow W. (New York, NY: Nova Science Publishers, Inc.), 145–176.

[B84] Winlow W. Polese G. Moghadam H. F. Ahmed I. A. Di Cosmo A. (2018). Sense and insensibility - appraisal of the effects of clinical anesthetics on gastropod and cephalopod molluscs as a step to improved welfare in Cephalopods. Front. Physiol. 9:1147. 10.3389/fphys.2018.01147 30197598 PMC6117391

[B85] Winlow W. Yar T. Spencer G. (1991). Studies on cellular mechanisms underlying general anesthesia using cultured molluscan neurons. Ann. NY Acad. Sci. 625, 269–272. 10.1111/j.1749-6632.1991.tb33846.x 2058886

[B86] Winlow W. Yar T. Spencer G. Girdlestone D. Hancox J. (1992). Differential effects of general anesthetics on identified molluscan neurons *in situ* and in culture. Gen. Pharmacol. 23, 985-992. 10.1016/0306-3623(92)90276-P 1487134

[B87] Wood G. Winlow W. (1996). Seasonal variation of spike width in identified neurones of *Lymnaea stagnalis*. J. Physiol. 495:38.

[B88] Yar T. (1992). The Effects of Halothane on Cultured Lymnaea Neurons. PhD Thesis, University of Leeds.

[B89] Yar T. Ahmed I. A. Winlow W. (1993). A simple computer program written in spike 2 script language to analyse single action potentials. J. Physiol. 468:137.

[B90] Yar T. Winlow W. (1991). Cultured Lymnaea neurones maintain their normal action potential types. J. Physiol. 434:58.

[B91] Yar T. Winlow W. (2016a). Isolation and characterization of whole-cell calcium channel currents in cultured, identified neurones of *Lymnaea. EC Neurol* 3.5, 449–458.

[B92] Yar T. Winlow W. (2016b). Effects of halothane on whole-cell calcium channel currents in cultured *Lymnaea* neurones. EC Neurol. 4, 3–22.

[B93] Zaitsev O. V. Shuvalova N. E. (1988). Morphological properties of neuron RPD1 in *Lymnaea stagnalis* and its involvement in processing polymodal sensory information. Neurophysiology 20, 571–578. 10.1007/BF02150261

